# Stat1 confers sensitivity to radiation in cervical cancer cells by controlling Parp1 levels: a new perspective for Parp1 inhibition

**DOI:** 10.1038/s41419-021-04229-y

**Published:** 2021-10-12

**Authors:** Giuseppina Raspaglio, Marianna Buttarelli, Flavia Filippetti, Alessandra Battaglia, Alexia Buzzonetti, Giovanni Scambia, Daniela Gallo

**Affiliations:** 1 Unità di Medicina Traslazionale per la Salute della Donna e del Bambino, Dipartimento Scienze della Salute della Donna, del Bambino e di Sanità Pubblica, Fondazione Policlinico Universitario A. Gemelli, IRCCS, Roma, Italia; 2 Dipartimento Universitario Scienze della Vita e Sanità Pubblica – Sezione di Ginecologia ed Ostetricia - Università Cattolica del Sacro Cuore, Roma, Italia; 3 Dipartimento Scienze della Salute della Donna, del Bambino e di Sanità Pubblica, Fondazione Policlinico Universitario A. Gemelli, IRCCS, Roma, Italia

**Keywords:** Cervical cancer, Mechanisms of disease

## Abstract

Cervical cancer (CC) is the fourth most common cause of cancer-related death in women. According to international guidelines, a standard treatment for locally advanced cervical cancer (LACC) consists of exclusive concurrent chemoradiation treatment (CRT). However, chemoradioresistance and subsequent relapse and metastasis of cancer occur in many patients, and survival for these women has generally remained poor. Therefore, strategies to overcome resistance are urgently needed. We have recently reported a radiosensitizing effect of the signal transducer and activator of transcription 1 (STAT1) in CC, associated with the control of [Poly(ADP-ribose) polymerase −1] PARP1 levels, a key factor in cell response to DNA damage induced by radiation. Here, we sought to decipher the underlying mechanism of STAT1-mediated control of PARP1, elucidating its role as a radiosensitizer in CC. Functional and molecular biology studies demonstrated that STAT1 may act at both transcriptional and posttranscriptional levels to modulate PARP1 expression in CC cells. In light of these results, we tested the effect of Olaparib in sensitizing CC cells to radiation and investigated signaling pathways involved in the activity observed. Results showed that PARP1 inhibition, at clinically achievable doses, may indeed selectively improve the sensitivity of resistant CC cells to DNA-damaging treatment. The translational relevance of our findings was supported by preliminary results in a limited patient cohort, confirming that higher PARP1 levels are significantly associated with a radioresistant phenotype. Finally, bioinformatics analysis of GEPIA and TCGA databases, demonstrated that PARP1 mRNA is higher in CC than in normal tissues and that increased PARP1 mRNA expression levels are associated with poor prognosis of LACC patients. Overall, our data open new opportunities for the development of personalized treatments in women diagnosed with CC.

## Introduction

Worldwide, cervical cancer (CC) is the fourth most frequent cancer in women, representing 7.7% of all female cancer deaths [1]. In LACC patients, exclusive concurrent chemoradiation is the preferred treatment [2–4]. However, recurrence rates remain high and survival in this clinical setting has generally remained poor [5], largely because of the intrinsic resistance of tumor cells to radiation. Therefore, strategies to overcome radioresistance are urgently needed.

We recently identified a biomarker signature able to predict response to neoadjuvant CRT in LACC patients and demonstrated that low levels of ANXA2 (annexin A2) and NDRG1 (N-myc downstream-regulated gene 1) and high levels of STAT1 are predictive biomarkers of sensitivity for patients receiving concomitant CRT [6].

STAT1 is one of the seven mammalian members of the STAT family and is best known for its essential role in mediating responses to all types of interferons (IFN), being preferentially activated by IFN-γ [7, 8]. STAT1 phosphorylation leads to its activation and nuclear translocation, although transcriptional activity has also been ascribed to unphosphorylated STAT1 (U-STAT1) [9]. Besides its involvement in antiviral and antibacterial response, STAT1 also induces growth inhibition and stimulation of apoptosis, thus suppressing tumor growth [8]. Nevertheless, conflicting data exist with respect to the role of STAT1 in determining responsiveness to DNA-damaging agents. Indeed, some reports link aberrant STAT1/IFN pathway activation with chemo- and radio-resistance [9, 10]. Conversely, other studies have shown that STAT1 actually promotes death after DNA damage [11–13]. In line with these latter evidences, we demonstrated that the functional consequences of *STAT1* silencing in radiosensitive (C-4I) or radioresistant (CaSki) CC cells were represented by a decrease in sensitivity to radiation and cisplatin, with increased clonogenicity observed in all treatment conditions. Our mechanistic investigations suggested that the radiosensitizing role of STAT1 could be at least partially linked to the control of PARP1 level in CC cells [6].

PARP1 is an abundant nuclear chromatin-associated protein, endowed with a high DNA damage-sensing ability. The protein, through the addition of poly-ADP-ribose (PAR) moieties to sites of single-strand DNA (ssDNA) damage, plays a critical role in DNA repair processes [14]. Notably, recent studies have supported the efficacy of various PARP inhibitors (PARP*i*) in radiosensitizing human cancer cell lines and xenograft models to ionizing radiation (IR) [15–17].

Here, we sought to investigate the underlying mechanism of STAT1-mediated control of PARP1 levels in CC cell lines, and its impact on determining the radiosensitizing effect observed in our previous study. Results demonstrated that STAT1 may act at both transcriptional and posttranscriptional levels to modulate PARP1 expression. To corroborate these findings, we tested the effect of Olaparib in sensitizing CC cells to IR and investigated signaling pathways involved in the activity observed. Overall, data obtained may move therapeutic advancement for the use of PARP*i* to improve tumor sensitivity to DNA-damaging agents in CC patients.

## Results

### The multifactorial role of STAT1-mediated control of PARP1 levels

It has been reported that STAT1 is able to regulate proteasome activity, by directly modulating proteasome regulators PA28α and PA28β [18]. Besides, very recent studies support the hypothesis that, in unchallenged conditions, PARP1 levels, are mainly regulated by proteasome degradation [19]. In light of these findings, and of our previous preliminary results suggesting a posttranscriptional control of PARP1 expression by STAT1 in CC cells [6], here we sought to decipher the underlying mechanism of this control.

**Fig. 1:**
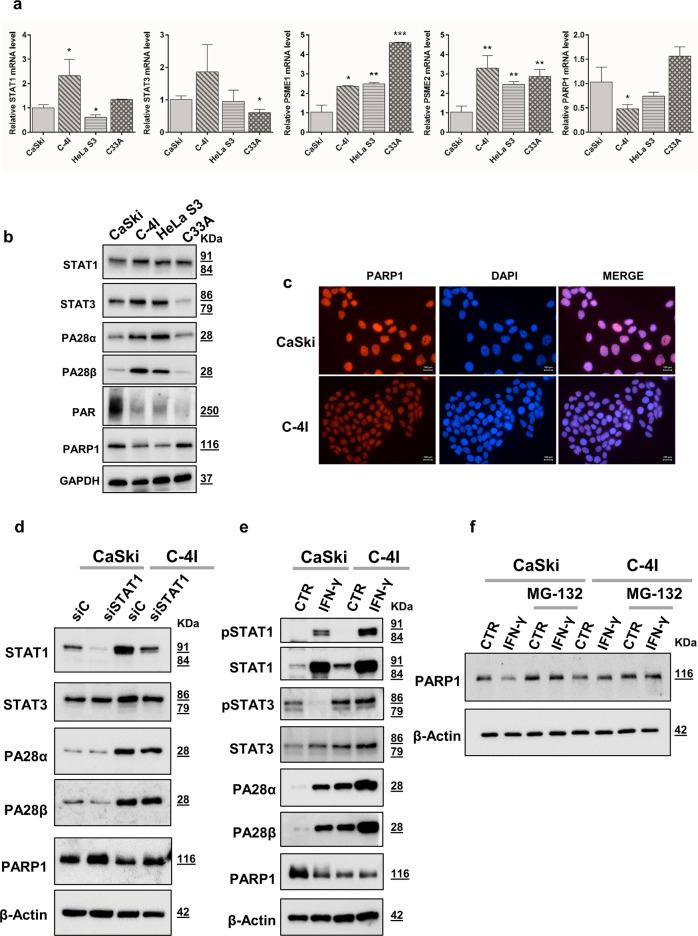
STAT1-mediated control of PARP1 levels in cervical cancer models. **a** Relative mRNA expression level of STAT1, STAT3, PSME1, PSME2, and PARP1 was evaluated by RT-qPCR in CaSki, C-4I, HeLa S3, and C33A cells. Samples were normalized to the mean of two housekeeping genes, GAPDH and PGK1. For each mRNA, results are presented as fold change compared to CaSki cells (mean ± SD, *n* = 3). Statistical significances have been evaluated through an unpaired *t*-test. *P < 0.05, **P < 0.01, ***P < 0.001 respect to CaSki cells. **b** Representative Western blot analysis of STAT1, STAT3, PA28α, PA28β, PARP1 proteins in CaSki, C-4I, HeLa S3, and C33A cells. GAPDH was used as a control. **c** Representative pictures showing immunolocalization of PARP1 in CaSki and C-4I cells (magnification 63x). **d**, **e** Representative Western blot of STAT1, STAT3, PA28α, PA28β, PARP1 proteins in CaSki, and C-4I cells. Cells were harvested 48 h following STAT1 silencing (**d**) or 10 ng/ml of IFN-γ treatment (**e**) and whole-cell lysates (10 μg) were loaded into SDS-PAGE, followed by Western blot with specific antibodies. β-Actin was used as a loading control. **f** CaSki and C-4I cells were treated with 10 ng/ml IFN-γ for 24 h and then added MG-132 (0.5 µM) for an additional 24 h. Cells were harvested and whole-cell lysates (10 μg) were loaded into SDS-PAGE, followed by Western blot with specific antibodies. β-Actin was used as a loading control. siC siRNA control, siSTAT1 siRNA targeted to STAT1, CTR untreated, IFN-γ interferon-γ. Data were representative of at least three experiments.

To this aim, a panel of CC cell lines, including three HPV16/18 positive (i.e., CaSki, C-4I, and HeLa S3) and one HPV negative (i.e., C33A) cell lines, was characterized in terms of expression of proteins of our interest, i.e., STAT1, PA28α/β, and PARP1 (along with PARP1 activation). Besides, due to the notion that STAT1 and STAT3 play opposite role in cancer-relevant processes [20], we also evaluated the expression of STAT3 in our experimental models (Fig. 1a, b). Results obtained showed that C-4I cells expressed the highest STAT1 levels (both mRNA and protein), while CaSki exhibited the lowest basal protein expression (Fig. 1a, b). On the other hand, lower STAT3 levels were observed in C33A, when compared to CaSki, C-4I, and HeLa S3. This latter finding is in line with previous studies showing that STAT3 protein expression (and phosphorylation) is increased in HPV positive compared to HPV negative CC cells [21]. Lower protein levels of both PA28 subunits, along with higher PARP1 levels, were observed in CaSki and C33A compared to C-4I and HeLa S3 cells. Finally, higher PARylation activity was found in CaSki in comparison to the remaining cell lines.

The radioresistant (CaSki) and the radiosensitive (C-4I) CC cells [6, 22] were then chosen to evaluate changes in PA28 activators and PARP1 levels, following modulation of STAT1 (i.e., gene silencing or pathway activation after treatment with IFN-γ). Immunofluorescence analysis confirmed different PARP1 expression between the two cell lines (Fig. 1c).

In both models, STAT1 silencing induced a decrease in PA28α and/or PA28β expression (Fig. 1d). These changes paralleled with increased PARP1 levels, mostly evident 48 h after gene silencing, in keeping with our previous results [6]. STAT1 silencing did not induce any relevant changes in the levels of STAT3 expression (Fig. 1d).

As expected, treatment with 10 ng/ml IFN-γ induced, both in CaSki and in C-4I, a significant increase in STAT1 levels with a strong protein activation, as evidenced by specific tyrosine phosphorylation at Tyr701 (Fig. 1e). Conversely, only a limited increase in STAT3 protein was appreciated at 48 h treatment (Fig. 1e). However, while in CaSki IFN-γ significantly reduced STAT3 activation [as evidenced by a decrease in the ratio between phosphorylated (Tyr705) and unphosphorylated forms], in C-4I no significant changes were observed. IFN-γ also significantly increased expression of PA28α and PA28β in both cell lines, a change accompanied by a decrease in PARP1 protein in CaSki, but not in C-4I (Fig. 1e). We may speculate that the failure in further decreasing PARP1 levels in C-4I, despite the increase in PA28 expression, could be the result of the vital widespread functions PARPs have in controlling cellular homeostasis [23].

Finally, treatment of control and IFN-γ- treated cells with a proteasome inhibitor (MG-132) confirmed the role of STAT1-mediated proteasome induction in regulating PARP1 levels. Indeed, as shown in Fig. 1f, MG-132 blocked the decrease in PARP1 levels induced by IFN-γ treatment in CaSki cells. In C-4I, a slight increase in PARP1 level after MG-132 treatment was evident in both control and IFN-γ-treated cells, without differences between the two.

**Fig. 2:**
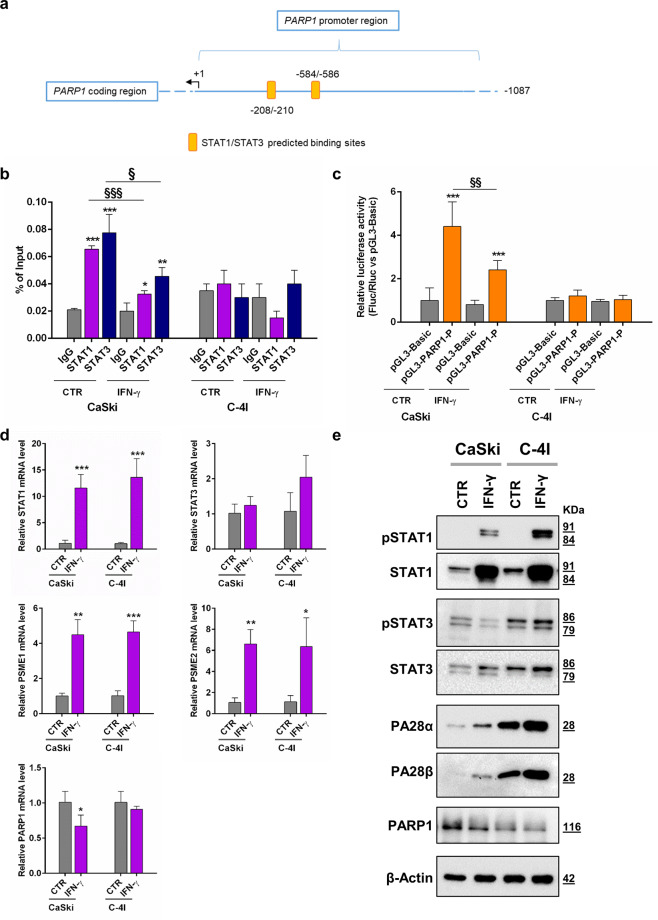
Transcriptional regulation of PARP1 by STAT1 in cervical cancer models. **a** Schematic presentation of the PARP1 gene promoter region analyzed using the MatInspector program. This region was investigated with ChIP-qPCR and inserted into the luciferase reporter gene construct (pGL3-PARP1-P). **b** Occupancy of STAT1 and STAT3 at the PARP1 promoter. Cells were treated with 10 ng/ml IFN-γ for 24 h and specific recruitment was assessed by ChIP-qPCR. The amount of precipitated DNA was calculated as percent of input (mean ± SD, *n* = 3). Statistical significances have been evaluated through an unpaired *t*-test. *P < 0.05, **P < 0.01, and ***P < 0.001, CTR or IFN-γ group with respect to IgG. ^§^*P* < 0.05 and ^§§§^*P* < 0.001 IFN-γ with respect to CTR. **c** Relative luciferase activity of PARP1 promoter in CaSki and C-4I cells. Vectors (pGL3-Basic or pGL3-PARP1-P) were transiently transfected with the pRL-TK vector (*Renilla* luciferase control reporter vector) as an internal control. After 6 h, 10 ng/ml IFN-γ was added and cells were analyzed after 24 h of treatment. Promoter activity is expressed as a ratio of Firefly to Renilla luciferase activity (Fluc/Rluc) normalized to the pGL3-Basic vector (mean ± SD, *n* = 5). Statistical significances have been evaluated through an unpaired *t*-test. ***P < 0.001 CTR or IFN-γ group with respect to pGL3-Basic. ^§§^*P* < 0.05 IFN-γ with respect to CTR. **d** Relative mRNA expression level was evaluated by RT-qPCR after 24 h of 10 ng/ml IFN-γ treatment. For each mRNA, samples were normalized to the mean of two housekeeping genes, GAPDH and PGK1 and results are presented as fold change compared to CTR cells. Statistical significances have been evaluated through an unpaired *t*-test. *P < 0.05, **P < 0.01, and ***P < 0.001, IFN-γ with respect to CTR group. **e** Representative Western blot analysis of proteins of interest in CaSki and C-4I cells. Cells were harvested 24 h following 10 ng/ml IFN-γ treatment and whole-cell lysates (10 μg) were loaded into SDS-PAGE, followed by Western blot with specific antibodies. β-Actin was used as a loading control. CTR untreated. IFN-γ interferon-γ. Data were representative of at least three experiments.

In order to explore the possibility that STAT1 could also directly regulate the expression of PARP1, we carried out an in silico analysis on genomic regions close to the PARP1 gene, using MatInspector. Results of this analysis (Fig. 2a) revealed two putative binding sites for STAT1 and STAT3 (score 0.85 and 0.94, respectively for both sites) in the proximal promoter region of the PARP1 gene, one site on the minus (STAT1) and on the plus (STAT3) strand [tctgtcccaGGAAgtctta, agacTTCCtgggacagaac, at −208/−210 from the transcriptional start site] and one again on the minus (STAT1) and on the plus (STAT3) strand [ctgggtccgGGAAgcgcag, gcgcTTCCcggacccagct, at −584/−586 from the transcriptional start site] on the 5′ flanking region of PARP1 gene.

To confirm the interaction of STAT1 and STAT3 with PARP1 promoter, we set up a ChIP-qPCR assay using CaSki and C-4I, in basal conditions and after 24 h of IFN‐γ treatment. Data obtained showed that in CaSki both STAT1 and STAT3 were bound to the PARP1 promoter and this binding was significantly reduced by IFN-γ (Fig. 2b). On the other hand, we did not observe any binding of STAT1/STAT3 in C-4I cells, neither in basal nor in stimulated conditions (Fig. 2b). To support these results, we carried out an additional ChIP-qPCR assay to verify the binding of STAT3 to PARP1 promoter in siSTAT1 C-4I cells. Interestingly we found that STAT1 silencing significantly increased STAT3 occupancy at the PARP1 promoter in this model (Supplementary Fig. 1).

A luciferase-based assay was then performed to investigate the IFN-γ/STAT1 pathway activation on PARP1 gene promoter activity. In CaSki, the PARP1 promoter construct showed significant activity compared with the negative control vector, and this activity was reduced by IFN-γ (Fig. 2c). On the other hand, in C-4I, the dual-luciferase assay showed a negligible promoter activity in basal conditions as well as after IFN-γ treatment (Fig. 2c), in line with the low endogenous PARP1 levels and with ChIP results showing a lack of binding of PARP1 promoter by STAT1/STAT3.

To correlate these findings with the expression of PARP1, we treated CC cells with 10 ng/ml IFN-γ for 24 h and then assessed changes in mRNA and protein levels. Importantly, IFN-γ determined a decrease in PARP1 mRNA and protein levels in CaSki cells, but not in C-4I (Fig. 2d, e), these findings being in line with results from Chip-qPCR and luciferase assays. Changes observed in STAT1/STAT3 levels (and activation) were in line with those observed 48 h after treatment (see above). Finally, as anticipated, treatment with IFN-γ also induced an increase in both mRNAs (PSME1 and PSME2) and protein PA28α and PA28β levels (Fig. 2d, e).

### IFN-γ/STAT1 pathway activation sensitizes radioresistant CC cell

To confirm the potentiation of radiation injury by STAT1, CC cells were exposed to 10 ng/ml IFN-γ, 2 h prior to IR. The IFN-γ was left in the medium for the duration of the experiment. Both radiation and IFN-γ treatments alone decreased the clonogenic survival in both CaSki and C-4I cells compared to untreated controls (Supplementary Fig. 2). As expected, CaSki were less sensitive to IR compared to C-4I, with survival fractions of 72.3 ± 2.7% and 56.3 ± 4.1% (mean ± SD), respectively. On the other hand, the two cell lines responded in a similar way to IFN-γ (Supplementary Fig. 2). With respect to the γ-rays alone, the combined treatment was able to further decrease cell survival in both cell lines to 21.7 ± 5.9% in CaSki cells and 27.4 ± 3.6% in C-4I (mean ± SD) (Supplementary Fig. 2). These results imply that the addition of IFN-γ to radiation actually abrogates the difference in sensitivity between radioresistant and radiosensitive CC models.

Finally, levels of viral E6 mRNA were measured in control and treated cells. Results showed that IFN-γ treatment was associated with a reduction in viral E6 in CaSki, but not in C-4I cells (Supplementary Fig. 3a, b). These findings are in line with previous data [24] showing that IFN-γ treatment induced a decrease in the level of HPV16/18 E6 transcripts in CaSki, but not in C-4II (C-4I and C-4II are distinct lines from the same cervical tumor). Interestingly, although E6 reduction was greater in CaSki than in C-4I cells, a comparable sensitivity to IFN-γ was detected, as evidenced by clonogenic survival fractions after treatment (Supplementary Fig. 2). Finally, IR alone did not induce any changes in both cellular models (Supplementary Fig. 3a, b).

### Olaparib sensitizes radioresistant CC cell

Having confirmed that the radiosensitizing effect of STAT1 may be mediated by PARP1, we investigated the effects of the PARP1-inhibitor Olaparib on clonogenic survival and radiosensitization of CaSki and C-4I cells.

**Fig. 3:**
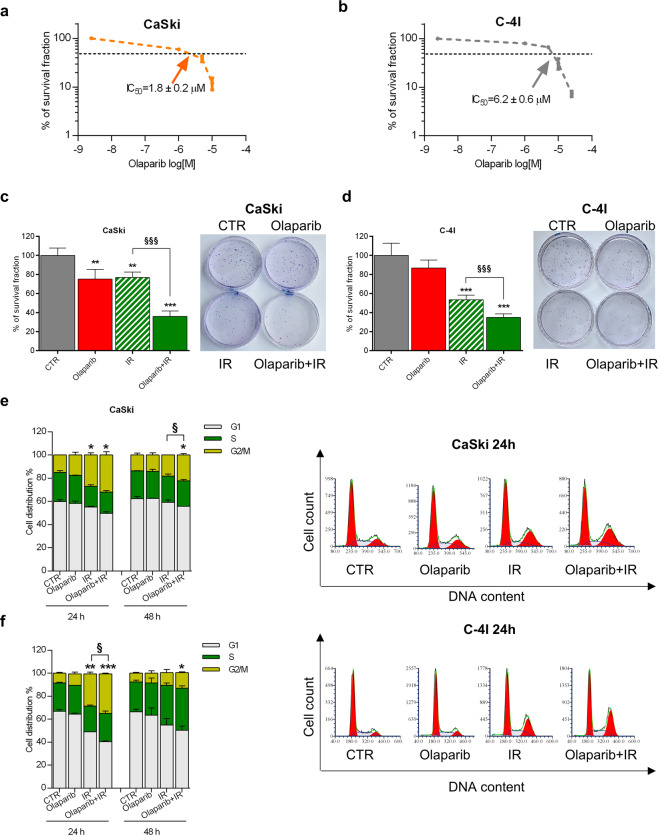
Olaparib sensitizes radioresistant cervical cancer cells. Clonogenic survival of CaSki (**a**) and C-4I (**b**) cells treated with Olaparib. Arrows indicate the IC50 value for each cell line (mean ± SEM). Clonogenic survival fractions of CaSki (**c**) and C-4I (**d**) cells treated with Olaparib with or without IR (2 Gy). For the combined treatments, the Olaparib dose that inhibited 30% of the colony-forming ability of CaSki (IC30, 0.7 µM) was added to the plates 1 h prior to gamma-irradiation. Cells were pretreated for 1 h with Olaparib and then irradiated. Bar charts represent the percentage of survival fraction normalized to CTR (mean ± SD, *n* = 4) and representative pictures are shown. Statistical significances have been evaluated through an unpaired *t*-test. **P < 0.01 and ***P < 0.001 respect to CTR. ^§§§^*P* < 0.001 combined treatment with respect to IR. Stacked percentages of CaSki (**e**) and C-4I (**f**) cells in the G1, S, and G2/M cell cycle phases after 24 and 48 h treatment of Olaparib (0.7 µM) with or without IR (2 Gy) (mean ± SD, *n* = 2). Cells were pretreated for 1 h with Olaparib and then irradiated. Representative plots of cell cycle analysis after 24 h of treatment are shown for each cell line. Statistical significances have been evaluated only for the G2/M phases through an unpaired *t*-test. *P < 0.05, **P < 0.01, and ***P < 0.001 respect to untreated cells (CTR). ^§^*P* < 0.05 combined treatment with respect to IR. CTR untreated. IR: 2 Gy γ-rays.

The CaSki cell line demonstrated to be more sensitive than C-4I to Olaparib (Fig. 3a, b). The combined treatment IR ± Olaparib, with respect to the IR alone, was able to further decrease cell survival in both cell lines, from 76.8 ± 5.8% to 36.1 ± 5.6% in CaSki and from 53.5 ± 5.0% to 34.9 ± 3.7% in C-4I (mean ± SD) (Fig. 3c, d). These results demonstrate that the co-treatment with PARP*i* enhances the radiosensitivity of resistant cells, also improving the effect of radiation in a sensitive model.

### Cell cycle and apoptosis analysis

In both cellular models, flow cytometry analysis did not show any modulation of distribution of cells in the different phases of the cell cycle at 24 and 48 h after treatment with Olaparib alone (Fig. 3e, f). Conversely, the combination IR/Olaparib induced an increase in the percentage of cells in the G2/M-phase 24 h after IR in C-4I cells, which lasted up to 48 h after treatment (Fig. 3f). A similar effect was observed in CaSki only at 48 h (Fig. 3e).

**Fig. 4:**
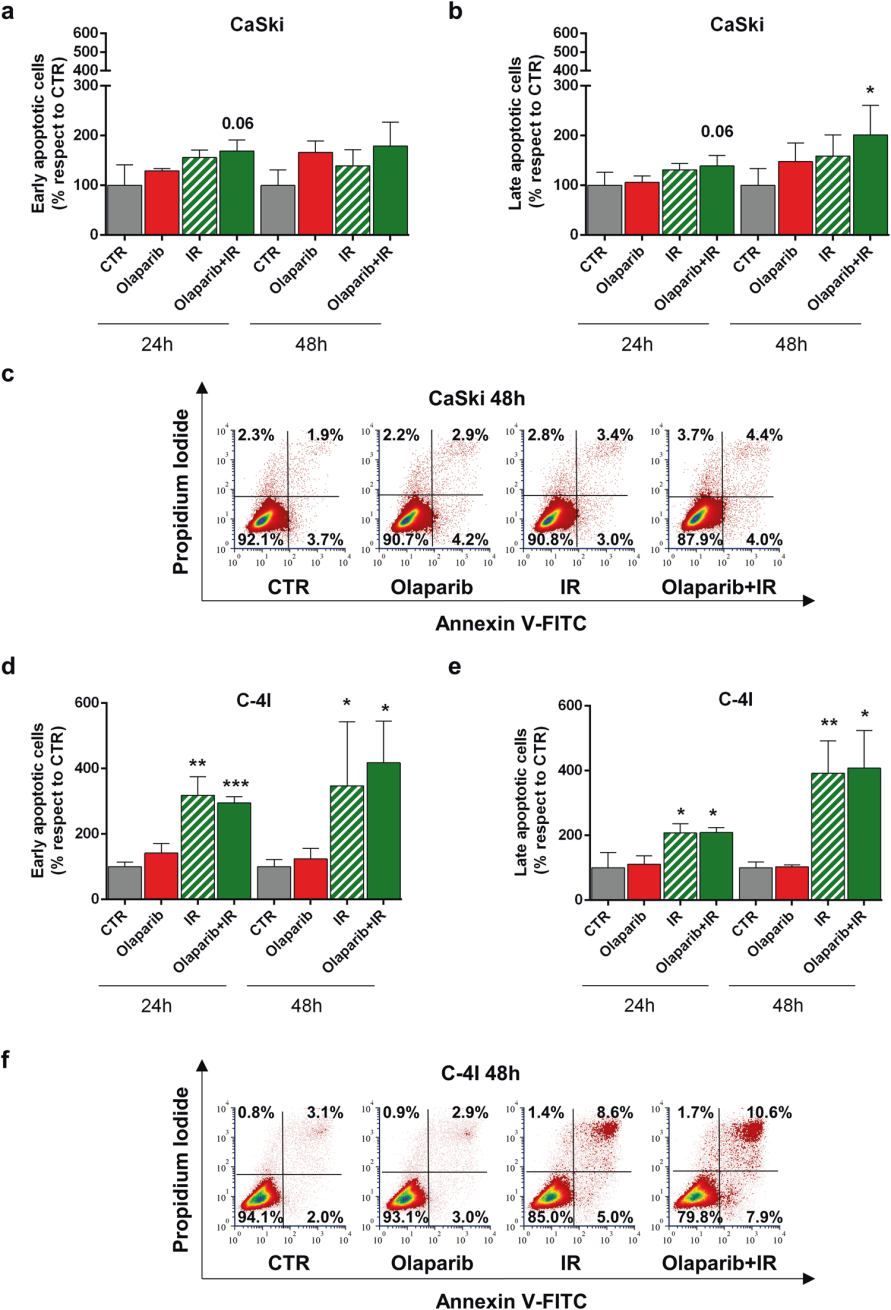
Olaparib effects on apoptosis of cervical cancer cells. Flow cytometry analysis of CaSki (**a–c**) and C-4I (**d–f**) cells stained with Annexin V and propidium iodide (PI). Bar graphs show percent variation of cells in early (Annexin V^+^/PI^−^, **a** and **d**) and late (Annexin V^+^/PI^+^, **b** and **e**) apoptosis with respect to CTR after 24 and 48 h treatment of Olaparib (0.7 µM) with or without IR (2 Gy) (mean ± SD, *n* ≥ 3). Cells were pretreated for 1 h with Olaparib and then irradiated. For each cell line, representative plots obtained by flow cytometric analysis at 48 h posttreatment are also shown (**c** and **f** Caski and C-4I cervical cancer cells, respectively). Statistical significances have been evaluated through an unpaired *t*-test. *P < 0.05, **P < 0.01, and ***P < 0.001 respect to CTR. CTR untreated.

The results of Annexin V/PI staining indicated that Olaparib alone did not trigger apoptosis either in CaSki or in C-4I cells (Fig. 4). Only minor changes in the percentage of apoptotic cells were observed in CaSki after 24/48 h of IR ± Olaparib (Fig. 4a, b). On the other hand, apoptosis was significantly induced 24 and 48 h following IR alone in C-4I cells with a further, slight increase associated with the addition of Olaparib (Fig. 4d, e). The modest increase in apoptosis observed in CaSki cells following the association of Olaparib to IR can only in part explain the cytotoxic effect of the combination highlighted by the clonogenic assay.

### Role of Olaparib in mediating radio-responsiveness

**Fig. 5:**
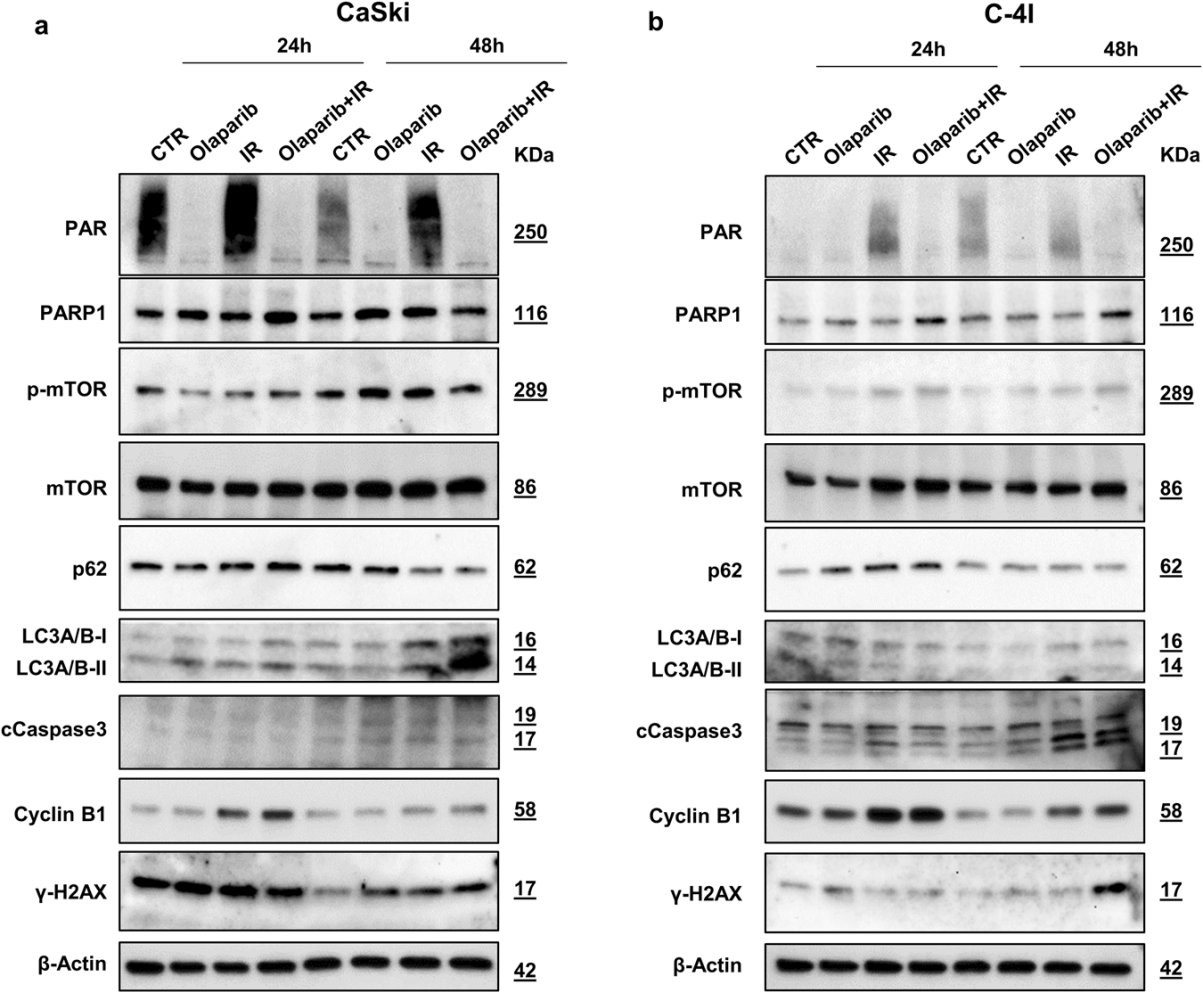
Modulation of protein levels by Olaparib with or without IR in cervical cancer cells. **a** CaSki and **b** C-4I cells were pretreated for 1 h with Olaparib (0.7 µM) and then irradiated (2 Gy). Cells were harvested 24 or 48 h after treatment and whole-cell lysates (30 μg) were loaded into SDS-PAGE, followed by Western blot with specific antibodies. β-Actin was used as a loading control. CTR untreated; IR 2 Gy γ-rays. Data were representative of at least three experiments.

In order to determine if inhibition of PARP1 enzymatic activity occurs at concentrations of Olaparib that are sufficient to induce radiosensitization, we treated cells with Olaparib ± 2 Gy radiation and measured the total PAR and PARP1 levels in CC cell lines. In CaSki cells, PAR formation was significantly inhibited with 0.7 μM Olaparib ± IR (Fig. 5a, b). A similar, although lower effect, was seen in C-4I cells. Although the drug effectively inhibits PARylation, the amount of PARP1 in the cell lines remains quite constant (Fig. 5a, b).

Western blot analysis showed that in CaSki cells the combined treatment triggered non-apoptotic, autophagy-dependent cell death, as evidenced by the increase in LC3 cleavage and by a decrease in p62 levels (Fig. 5a) [25]. Autophagy was mostly evident after 48 h in the co-treatment condition, although a slight increase was also found in irradiated (IR)-only cells. The conspicuous increase of autophagy caused in CaSki cells by the co-treatment was associated and reasonably generated, by a fall of Ser-2448 phosphorylated mTOR kinase (Fig. 5a). In C-4I cells, upregulation of Cyclin B1 closely matched with the Olaparib/IR-induced G2 cell cycle arrest, in line with our previous results demonstrating that mitotic catastrophe (characterized by a significant G2/M cell cycle arrest) is a major form of IR-induced cell death in C-4I, but not in CaSki [6]. Besides, the combination of Olaparib and radiation induced an increase in γH2AX foci formation. These events were accompanied by apoptosis, as evidenced by cleaved caspase-3 protein, showing an increase after IR with a further rise associated with the addiction of Olaparib (Fig. 5b).

Overall, results from functional and molecular biology studies suggest that in CaSki, Olaparib induces radiosensitization primarily driving cells to autophagy, this being reasonably followed, at later times, by apoptosis, as shown by other authors [26]. On the other hand, in C-4I, the slight enhancement of the cytotoxic effect of radiotherapy by adding a PARP inhibitor is mainly due to persistent DNA damage.

### Differential expression of PARP1 in sensitive and resistant LACC patients

**Fig. 6:**
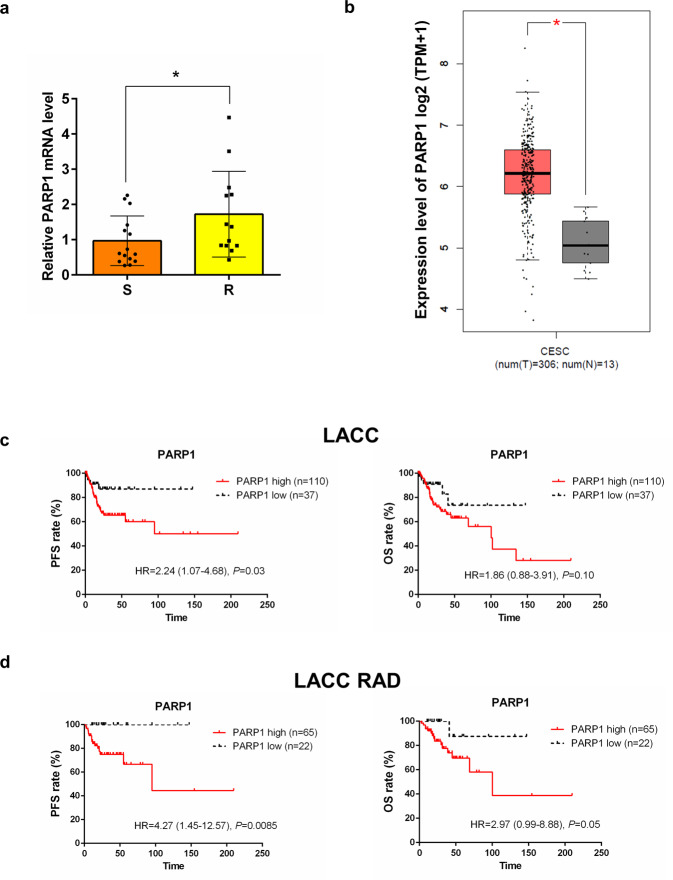
PARP1 expression and prognosis in cervical cancer patients. **a** Relative PARP1 mRNA expression was evaluated by RT-qPCR and samples were normalized to B2M. The relative expression of PARP1 was calculated with the ΔΔCt method, using the mean ΔCt of all samples as a reference sample (S = 15, R = 13). Statistical significances have been evaluated through an unpaired *t*-test. *P < 0.05 respect to sensitive patients. **b** Gene expression analysis of PARP1 using GEPIA (gene expression profiling interactive analysis) database (http://gepia.cancer-pku.cn/) based on the TCGA and GTEx database. Box plots represent the gene expression level in terms of log2 (TPM + 1) in the tumor (CESC cervical squamous cell carcinoma and endocervical adenocarcinoma, red, *n* = 306) and normal (gray, *n* = 13) samples, respectively.*P < 0.05. **c**, **d** Kaplan–Meier survival curves for the probability of PFS (progression-free survival) and OS (overall survival) in TGCA-CESC patients cohort selected for locally advanced cervical cancer (LACC, **c** *n* = 147) and for LACC and radiation therapy (**d** *n* = 87). PARP1 expression values were converted into discrete variables by dividing the available population cohorts into “high PARP1” and “low PARP1” using the 25th percentile as cutoff: **c** PARP1 Cutoff = 3906,42; **d** PARP1 Cutoff = 3837,37. *P* value in the plot represents the result of the log-rank test.

To corroborate our preclinical findings on the role of PARP1 in mediating radioresistance in CC, we measured its mRNA levels in pretreatment biopsies from a limited cohort of sensitive (*n* = 15) and resistant (*n* = 13) LACC patients, selected as previously reported [6]. Notably, results obtained showed that higher PARP1 levels were significantly associated with a radioresistant phenotype (Fig. 6a). This finding, however, requires further confirmation in a larger cohort.

### Differential expression of PARP1 between CC and normal tissue

Results obtained from the GEPIA dataset provided strong evidence that the PARP1 level is upregulated in cancer compared to normal tissues (Fig. 6b), in line with previous data reporting increased copy overexpression of PARP1 in CC and supporting a role for the protein in the malignant progression of the disease [27].

### Prognostic role of PARP1 in LACC patients

Finally, to investigate a possible role for PARP1 as a prognostic biomarker in CC patients we interrogated the TCGA dataset through cBioportal and evaluated the association between mRNA levels and the risk of recurrence or death. We decided to limit analysis only to LACC patients (Table 1), whose standard treatment is concomitant chemoradiotherapy. Results obtained showed that PARP1 is a strong prognostic factor for PFS (Fig. 6c). After restricting the analysis to patients treated with radiation, a significant association was found between PARP1 levels and outcome, in terms of both PFS and OS (Fig. 6d).

**Table 1:** Clinicopathological characteristics of TGCA-CESC patient cohort.

Characteristics	LACC patients no. (%)	LACC RAD patients no. (%)
All cases	147	87
Histotype		
Squamous	128 (87.1)	75 (86.2)
Adenosquamous/Adenoma	19 (12.9)	12 (13.8)
Age (years)		
Median, range	49 (20–85)	48 (20–79)
Radiation		
Yes	87 (59.2)	87 (100)
No	18 (12.2)	-
NA	42 (28.6)	-
FIGO stage		
IB2-IIA2	60 (40.8)	31 (35.6)
IIB	39 (26.5)	25 (28.7)
III	41 (27.9)	25 (28.7)
IVA	7 (4.8)	1 (1.1)
Grade		
1–2	72 (49.0)	38 (43.7)
3	59 (40.1)	40 (46.0)
4	1 (0.7)	1 (1.1)
NA	15 (10.2)	8 (9.2)

*NA*, Not available.

## Discussion

By elucidating the mechanisms underpinning STAT1-mediated control of PARP1 levels, results from the present study provide further support to our previous data on the radiosensitizing role of STAT1 in CC [6]. To the best of our knowledge, this is the first report describing this level of crosstalk/interactions between STAT1 and PARP1.

Previous studies reported an implication of STAT1 in the DNA damage response (DDR), with STAT1 activation observed upon exposure of tumor cells to genotoxic insult [11, 28, 29]. Interestingly, a recent study demonstrated how induction of IFN-related genes is an early event that discriminates chemosensitive from chemoresistant tumors, with both P-STAT1 and U-STAT1 possibly playing a role in activating gene transcription after chemotherapy in vivo [30]. Findings from the present study are in keeping with these literature data, supporting a model in which STAT1 may improve the efficacy of DNA-damaging treatments, at least partially, by lowering PARP1 levels in tumors. Notably, our data suggest that both transcriptional and posttranscriptional mechanisms might be involved in the role of STAT1-mediated control of PARP1 levels. Specifically, with regard to the posttranscriptional control, we demonstrated that the high basal STAT1 levels in C-4I cells are associated with high PA28α/β expression [known to be associated with high proteasome proteolytic activities [31]], which, in turn, could keep PARP1 levels low. On the other hand, the low STAT1 expression in CaSki, with related negligible PA28α/β levels, accounts for higher PARP1 levels. Our data also suggest that low levels of U-STAT1, along with constitutive STAT3 activation, like occurs in CaSki, may allow STAT3 to drive transcriptional outputs, by working singly (homodimers) and/or cooperatively (heterodimers with STAT1), to sustain PARP1 gene transcription. In support of this hypothesis, our results indicate that the IFN-γ-induced suppression of pY-STAT3 in CaSki is associated with reduced binding of both STAT1 and STAT3 to the PARP1 promoter, and, in turn, to a decreased activity of the promoter itself. In this context, it is worthy to mention findings from Hirahara and colleagues [32], demonstrating that much of STAT1 binding to chromatin is STAT3-dependent and STAT3 is responsible for transcriptomic output drive, whereas STAT1 mainly plays a regulatory role. In line with this idea, we could speculate that, in C-4I cells, excess STAT1 sequesters STAT3 in the cytoplasm, thereby blocking its action, as also previously reported in other experimental models [33]. Accordingly, we found that STAT1 silencing in C-4I cells induced STAT3 occupancy at the PARP1 promoter.

To corroborate these findings, we investigated PARP1 inhibition as a radiosensitization strategy in CC, showing that the use of PARP1*i* as radiosensitizing agents is meaningful from a biological perspective and has the potential to be relevant at a clinically achievable dose. These data are in line with evidences demonstrating hypersensitivity to γ-irradiation in different PARP1-depleted cancer cell lines [34, 35]. Interestingly we found that the addition of Olaparib at the clinical 2 Gy radiation dose overcomes the difference in sensitivity between radioresistant and radiosensitive cell models and that short exposure and very low dose levels are sufficient to achieve the desired effect. Likewise, Shunkwiler et al. [36] demonstrated substantial gains in cytotoxicity combining a 2 Gy clinical radiation dose to Veliparib. Notably, we observed that in CaSki the radiosensitizing effect was associated with significant autophagy induction in cells treated with the combined treatment. Mechanistically, our study has revealed a decreased p-mTOR/mTOR ratio following the combo treatment, in line with previous findings on PARP inhibitor-induced autophagy [37], and with studies linking the mTOR pathway and endoplasmic reticulum (ER) stress to radiation-induced cell death by autophagy [38]. Indeed, although autophagy is usually a cytoprotective process, autophagic cell death has been shown to occur when the autophagic response is excessive [39, 40]. On the other hand, in C-4I, a combination of Olaparib and radiation mainly induce γH2AX foci formation and cell apoptosis, in keeping with other data [41].

The translational relevance of our findings was supported by preliminary results obtained in a limited cohort of patients confirming that higher PARP1 levels are significantly associated with a radioresistant phenotype. Besides, bioinformatics analysis of publicly available datasets, in line with previous data [42], showed higher expression of PARP1 in primary CC compared to normal tissues and evidenced the role of PARP1 as a prognostic biomarker in LACC patients. In this context it is worthy to mention findings from Bianchi and colleagues [43], strongly supporting the idea that PARP1 is a good therapeutic target in CC and that a high level of PARylation may represent a useful biomarker for the identification of patients benefiting the most from PARPi [43, 44]. Currently, a Phase I/II Study of Niraparib with radiotherapy for treatment of metastatic invasive carcinoma of the cervix (NCT03644342) is recruiting patients. Besides CC, the concept of PARP inhibitor-mediated radiosensitization is being explored in many other cancer contexts [45]. However, many questions still remain unresolved, including effective doses, mechanism of action, and biomarkers of response.

Overall our results show that STAT1 control PARP1 levels through multiple mechanisms, possibly involving also STAT3. Besides, we also add evidence to the use of PARPi as an effective therapeutic approach to improve tumor sensitivity to DNA-damaging agents. These findings hopefully open new future perspectives for personalized treatment strategies in LACC patients.

## Materials and methods

### Cell culture and transfections

CaSki, C-4I, and HeLa S3 cells (ECACC) were purchased from Sigma-Aldrich (Darmstadt, Germany). Dr. Marco Paggi (IRCCS-Regina Elena National Cancer Institute) donated the C33A [46]. CaSki and C-4I were maintained as previously described [6]. HeLa S3 and C33A were cultured in Dulbecco’s modified Eagle’s medium (DMEM, Sigma-Aldrich) supplemented with 10% fetal bovine serum, 1 mM glutamine, 1% MEM non-essential amino acid, and 1% kanamicin. Cells were routinely tested for the absence of mycoplasma with the MycoAlert kit (LONZA, 169 Rockland, ME, USA).

Predesigned SMARTpool siRNAs targeting *STAT1* and nontargeting control siRNA (siC) were purchased from Dharmacon (Lafayette, CO, USA). TransFectin Lipid Reagent (Bio-Rad Laboratories, Hercules, CA) was used for transfection experiments as suggested by the supplier.

### Ionizing radiation and treatments

Cells were IR with an IBL 437 C γ-irradiator (Schering, Gir-Sur-Yvette Cedex, France) provided with a Cesium^137^ source and a dose rate of 2.05 Gy/min. We chose to use a single 2 Gy dose, since tumor radiosensitivity around 2 Gy has been proposed as a marker for tumor radiocurability, at least for those protocols that use multiple fractions in this dose range [47].

IFN-γ (Sigma-Aldrich) was dissolved in distilled water. For combination studies, 2 h before irradiation, cells were preincubated with either IFN-γ (10 ng/ml) or distilled water, and then IR with a 2 Gy dose. Selection of dosage and time of exposure was made based on previous literature data [48, 49] and preliminary internal studies (data not shown).

Olaparib (Cayman Chemical, Ann Arbor, Michigan, USA) was dissolved in DMSO. For combination studies, 1 h before irradiation, cells were preincubated with either Olaparib or DMSO, and then IR with a 2 Gy dose.

MG-132 (Sigma-Aldrich, dissolved in DMSO), a proteasome inhibitor, was added at 0.5 μM 24 h later IFN-γ treatment and cell harvested after further 24 h.

### In silico analysis

Genomic sequences upstream of PARP1 gene proximal promoter (−1087 from the transcriptional start site) were screened for putative transcription factor binding sites identification using Genomatix software suite v3.12, MatInspector program (http://www.genomatix.de, Genomatix Software GmbH, Munich, Germany) [50]. Parameters used for analysis were set as follows: Matrix Library 11.2, core similarity 0.75, and optimized matrix similarity.

### Chromatin immunoprecipitation (ChIP) PCR assay

The chIP-PCR assay was carried out to investigate the involvement of STAT1/STAT3 putative binding sites on the regulation of PARP1 expression levels. CaSki and C-4I cells treated, or left untreated, for 24 h with IFN-γ or C-4I cells silenced with siC or siSTAT1 for 48 h, were fixed in 1% formaldehyde containing PBS (10 min, room temperature), and the reaction stopped by addition of glycine 125 mM. After washing with PBS, cells were scraped, collected by centrifugation, and nuclei were isolated by incubating cells in hypotonic buffer (10 mM HEPES/KOH pH 7.5, 10 mM EDTA, 0.5 mM EGTA, 0.25% Triton X-100). Nuclei were collected by centrifugation and resuspended in lysis buffer [1%SDS, 10 mM EDTA, 50 mM Tris/HCl (pH 8.1)] plus protease and phosphatase inhibitors. Genomic DNA was sonicated on ice to fragments of 0.5–1 kb and debris was removed by centrifugation. Ten micrograms of precleared chromatin were incubated with 0.6 μg of either anti-STAT3 monoclonal antibody (124H6, Mouse monoclonal antibody #9139, Cell Signaling Technology, Danvers, MA) or anti-STAT1 polyclonal antibody (Stat1 Rabbit polyclonal antibody #9172, Cell Signaling Technology), or an anti-mouse or anti-rabbit IgG for negative control (Bio-Rad Laboratories, Hercules CA) for 16 h at 4 °C with rotation. Of the total input chromatin, 2% was not immunoprecipitated and used as a reference to express qPCR data. Then 30 μl protein A/G agarose slurry (sc-2003, Santacruz Biotechnology, Dallas, TX) was added to the mixture and incubated for 2 h at 4 °C with rotation. The complexes were isolated by microcentrifugation at 100 x *g* for 30 s. Pellets were washed three times (5 min per wash) on a rotating platform with buffer containing 150 mM NaCl, 2 mM EDTA, 20 mM Tris/HCl pH 8, 1% Triton X-100, and 1 time with buffer containing 500 mM NaCl, 2 mM EDTA, 20 mM Tris/HCl pH 8, 1% Triton X-100. After the last wash, immunocomplexes were eluted in freshly prepared elution buffer (1% SDS, 50 mMNaHCO3). Input chromatin samples and immunoprecipitations were mixed with NaCl at the final concentration of 200 mM and protein–DNA crosslinks were reverted by incubating the mixture at 65 °C for 16 h in a shaking thermomixer in the presence of proteinase K. DNA was purified using the Qiaquick PCR purification kit (Qiagen, Hilden, Germany). Genomic regions close to the putative STAT1/STAT3 binding sites were qPCR amplified using the following primers: forward: 5′-CCTGTAGTCCCAGCTACTC-3′, reverse: 5′-CCTGATAGATTGCTGATGC-3′ comprising both putative binding sites. The qPCR program was adapted for “GC” rich long amplicons as follows: 15′ at 95 °C, 40 cycles with 2 min 95 °C, 20 sec at 60 °C, and 20 sec at 72 °C, using the SsoFast Advanced universal SYBR Green mastermix (Bio-Rad) with 7% DMSO. Results were expressed as a percentage of input DNA using the formula: Percent input method [100 * 2^^(Adjusted input to 100% − Ct (IP)^].

### Luciferase assay

PARP1 (NM_001618) promoter region (−1087 + 1) was synthesized by GeneScript (GeneScript Corporation, NJ, USA) and subcloned into a pGL3 firefly basic vector. To assess luciferase activity, pGL3 firefly luc as an experimental reporter and pRL-TK renilla luc as an internal control were used. CaSki and C-4I cells were transfected with the described reporter vectors together with the renilla luciferase normalization plasmid (pRL-TK), using TranSfectin Lipid Reagent (Bio-Rad). After 6 h of transfection, cells were treated with IFN-γ (10 ng/ml) or distilled water, and luciferase activity was measured 20 h later using the Dual-Luciferase Reporter assay system (Promega, Madison, WI) according to the manufacturer instructions.

### Clonogenic assay

The effects on cell survival following IFN-γ or Olaparib as single or combined treatment with radiation were evaluated by a clonogenic assay. CaSki and C-4I cells were seeded in 60 mm diameter Petri dishes at a density of 500/dish. The day after plating, cells were IR, with or without a previous incubation of 2 h IFN-γ (10 ng/ml). A dose-response curve was generated for Olaparib treatment and the corresponding IC50 value was determined for each cell line. For the combined treatments, the Olaparib dose that inhibited 30% of the colony-forming ability (IC30) of CaSki was added to the plates 1 h prior to gamma-irradiation. Eight to 10 days after IR, surviving colonies with more than 50 cells were counted after fixation with ice-cold methanol and staining with 0.5% w/v crystal violet. Normalization to untreated control in each condition allowed to calculate the plating efficiency (PE), defined as the number of colonies counted/number of cells plated × 100 [51]. The surviving percentage was expressed as [*n*° of colonies in treated sample/(*n*° of plated cells × PE/100)] × 100.

### Cell cycle analysis by flow cytometry

Cells were treated with Olaparib/DMSO ± IR, as described above. At the end of each incubation period, adherent cells were trypsinized, harvested, and washed several times with cold Phosphate-buffered saline (PBS). Cells were then counted, gently fixed in 70% v/v cold ethanol, and incubated at −20 °C for no longer than 7 days. Prior to DNA staining, fixed cells were spun down and treated with RNase (100 μg/ml) for 10 min to ensure that only DNA was stained. Then, 1 × 10^6^ cells/ml were stained with propidium iodide (PI; 0.05 mg/ml) and stored at 4 °C overnight. The day after, stained cells were subjected to flow cytometry for cell cycle analysis by quantitation of cellular DNA content using the Beckman Coulter Navios flow cytometer (Brea, CA, USA). A minimum of 30,000 cells of interest were acquired for each sample, at a low flow rate (<200 events/sec). Analysis of cell cycle perturbation was performed by the Multicycle AV DNA analysis available in the FCS Express 7 software (De Novo Softwares Pasadena, CA, USA). Pulse shape processing was used to exclude cell doublets from the analysis.

### Flow cytometry analysis of apoptosis

Cells were treated with Olaparib/DMSO ± IR, as described above. Annexin V-fluorescein isothiocyanate (FITC)/PI (BD Biosciences, San Diego, CA) double staining method was used to detect cells undergoing early and late apoptosis. Briefly, at the end of treatments both adherent and floating cells were harvested and suspended at 1 × 10^6^ cells/ml in 1X binding buffer (10 mM HEPES/NaOH pH 7.4, 140 mM NaCl, and 2.5 mM CaCl_2_). Then, 5 µl of Annexin V-FITC and 1 µl PI (final concentration 1 μg/ml) were added, and cells were incubated for 15 min in the dark. The double-stained cells were analyzed by flow cytometry using the Beckman Coulter Navios flow cytometer within 30 min from staining and data collected and analyzed using the FCS Express 7 software. The binding of Annexin V to cells with the integer cell membrane (i.e., in the absence of PI co-staining) was used as a marker of early apoptosis. The binding of Annexin V to cells with the non-integer cell membrane (i.e., positively co-staining for PI) was used as a marker of late apoptosis.

### Real-time quantitative PCR

Real-time qPCR on mRNAs was performed as previously described [52] using the primers listed in Table 2. The geometric mean of GAPDH and PGK1 was taken as reference gene, following the GeNorm algorithm [53]; relative quantification of target mRNA was performed according to the ΔΔCt method [54]. For tumor tissue analysis, samples were amplified in triplicate and normalized to the housekeeping gene, B2M. The relative expression of PARP1 was calculated with the ΔΔCt method [54], using the mean ΔCt of all samples as a reference sample (S = 15, R = 13).

**Table 2:** Primer sequences used for RT-qPCR.

Gene symbol	Primer forward	Primer reverse	Amplicon length (bp)
*STAT1*	ATGCTGGCACCAGAACGAA	GCTGGCACAATTGGGTTTCAA	84
*STAT3*	GAGAAGGACATCAGCGGTAA	CAGTGGAGACACCAGGATAT	177
*PA28α*	CCACACCAAGCTAGAAGGCT	AGCATTGCGGATCTCCATGAC	138
*PA28β*	CTTTTCCAGGAGGCTGAGGAAT	ATCATCCTTGGGTGGAGGGT	158
*PARP1*	GCCCTAAAGGCTCAGAACGA	CTACTCGGTCCAAGATCGCC	141
*GAPDH*	GAACGGGAAGCTTGTCATCAA	ATCGCCCCACTTGATTTTGG	79
*PGK1*	GTGGAATGGCTTTTACCTTCC	CTTGGCTCCCTCTTCATCAA	60
*B2M*	TTAGCTGTGCTCGCGCTAC	CTCTGCTGGATGACGTGAGTAA	90
HPV16 *E6*	GTATGGAACAACATTAGAACAGCAA	GTGGCTTTTGACAGTTAATACACC	79
HPV18 *E6*	AAGATTTATTTGTGGTGT	GCTGGATTCAACGGTTTC	196

### Western blot analysis

Western blot analysis of total cell lysates was performed as previously described [6]. Table S1 shows the list of antibodies used. After incubation with secondary horseradish peroxidase-conjugated antibodies (Bio-Rad), specific proteins were visualized by the enhanced chemiluminescence system using a ChemiDoc^™^ XRS + imaging system (Bio-Rad).

### Fluorescence microscopy

Fluorescence microscopy was performed as previously described using an anti-PARP1 antibody (1:800, 46D11, Cell Signaling Technology) [6]. Slides were observed under a fluorescence microscope (Carl Zeiss microscopy Gmbh, Jena, Germany), using a 63X oil immersion objective.

### Patients and samples collection

The patient cohort used in this study was previously described and all patients had signed a written informed consent agreeing to submit to all the procedures defined and with their data to be collected (Protocol study approved by Ethics Committee of Policlinico Universitario. A. Gemelli P/966/CE/2012) [6]. In the present study, we analyzed PARP1 mRNA expression from 15 Sensitive (**S**, i.e., pathological complete response) and 13 Resistant (**R**, i.e., macroscopic residual tumor) LACC patients, selected as previously reported [6].

### Bioinformatics analysis of TCGA database

Cervical squamous cell carcinoma (CESC) data were retrieved from the publicly available and curated database cBioPortal [www.cbioportal.org, 2020 (accessed 1 June 2020)], extracting clinical and gene expression features from the TCGA PanCancer Atlas dataset [55, 56]. Gene expression levels were obtained from the mRNA Expression RSEM (Batch normalized from Illumina HiSeq_RNASeqV2) dataset (CESC, TCGA, PanCancer Atlas) and matched to the clinical features. Samples with insufficient information (gene expression values and survival information) were excluded from the analysis. Patients with LACC (FIGO stage IB2 through stage IVA) or with LACC and radiation treatment (LACC RAD) were selected for the analysis. PARP1 expression values were converted into discrete variables by dividing the available population cohorts into “high PARP1” and “low PARP1” using the 25th percentile as cutoff: (LACC) PARP1 Cutoff = 3906,42; (LACC RAD) PARP1 Cutoff = 3837,37. The prognostic effect of PARP1 levels on PFS (progression-free survival) and OS (overall survival) probabilities was estimated using the Kaplan–Meier method and survival curves were evaluated using the log-rank test. Statistical analysis was performed using Stata software (StataCorp. 2011. Stata Statistical Software: Release 12. College Station, TX: StataCorp LP). The gene expression profiling interactive analysis (GEPIA) dataset (http://gepia.cancer-pku.cn), based on The Cancer Genome Atlas (TCGA) and the GTEx project was used to compare PARP1 expression levels in CC and normal tissues [57].

### Statistical analysis

Means and standard deviation (SD) were calculated for all data points, from at least three independent experiments. Dose-response curve-fit was calculated with v6.0 GraphPad Prism (GraphPad Software, La Jolla, CA, USA). Unpaired Student’s *t*-test (or unpaired *t*-test with Welch’s correction if unequal variances) was used to analyze and compare the means. A statistically significant difference was considered when *P* ≤ 0.05.

## Supplementary information

Supplemental Material

## Data Availability

All data generated or analyzed during this study are included in this published article [and its supplementary information files]. The datasets analysed during the current study are available in cBioPortal [www.cbioportal.org, 2020 (accessed 1 June 2020)] and GEPIA dataset (http://gepia.cancer-pku.cn).
