# Supplementary material for: Stat1 confers sensitivity to radiation in cervical cancer cells by controlling Parp1 levels: a new perspective for Parp1 inhibition
Source: Cell Death Dis. 2021 Oct 12;12(10):933. doi: 10.1038/s41419-021-04229-y (PMC8511191; doi:10.1038/s41419-021-04229-y)
Supplement: Supplementary file 1 — Supplemental Material [file 41419_2021_4229_MOESM1_ESM.docx]

**SUPPLEMENTAL MATERIAL**

**STAT1 CONFERS SENSITIVITY TO RADIATION IN CERVICAL CANCER CELLS BY CONTROLLING PARP1 LEVELS: A NEW PERSPECTIVE FOR PARP1 INHIBITION**

*Raspaglio et al.*

This Supplemental Material contains:

**Supplementary Table 1**: List of Antibodies used in Western Blot analysis

**Supplementary Figure 1**: STAT1-silencing increases STAT3 occupancy at the PARP1 promoter

**Supplementary Figure 2**: Interferon-γ sensitizes radioresistant cervical cancer cells

**Supplementary Figure 3:** Effects of Interferon-γ, with or without IR, on viral E6 mRNA in cervical cancer cells

**Supplementary Table 1.** List of Antibodies used in Western Blot analysis.

| Antibody name | Clone | Supplier |
| --- | --- | --- |
| Phospho STAT1 (Tyr701) | Polyclonal | Santa Cruz Biotechnology (Santa  Cruz, CA) |
| STAT1 | HPA000931 | Sigma-Aldrich (Darmstadt, Germany) |
| PA28α | AT12H3 | Santa Cruz Biotechnology |
| PA28β | G-10 | Santa Cruz Biotechnology |
| Poly(ADP)-ribose (PAR) | 10H | Santa Cruz Biotechnology |
| PARP1 | 46D11 | Cell Signaling Technology (Boston,  MA) |
| Phospho STAT3 (Tyr705) | D3A7 | Cell Signaling Technology |
| STAT3 | 124H6 | Cell Signaling Technology |
| Phospho mTOR (Ser2448) | Polyclonal | Cell Signaling Technology |
| mTOR | 7C10 | Cell Signaling Technology |
| p62 (SQSTM1) | D3 | Santa Cruz Biotechnology |
| LC3A/B | D3U4C | Cell Signaling Technology |
| Cleaved Caspase-3 | 5A1E | Cell Signaling Technology |
| Cyclin B1 | Y106 | Abcam (Cambridge, UK) |
| γH2AX (S139) | Polyclonal | Cell Signaling Technology |
| β-Actin | AC-15 | Sigma-Aldrich |
| GAPDH | 6C5 | Abcam |

**Supplementary Fig. 1**

**
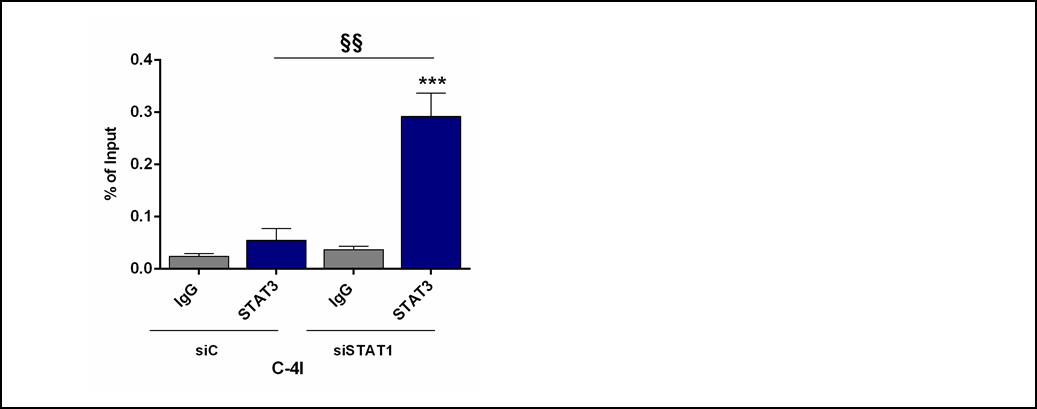
**

**Supplementary Fig. 1 STAT1-silencing increases STAT3 occupancy at the PARP1 promoter.** C4-I cells were silenced for STAT1 for 48h and specific STAT3 recruitment was assessed by ChIP-qPCR. The amount of precipitated DNA was calculated as percent of input (mean ± SD, n=3). Statistical significances have been evaluated through an unpaired t-test. ***P<0.001 siSTAT1 group with respect to IgG. §§P<0.01 siSTAT1 with respect to siC.

**Supplementary Fig. 2
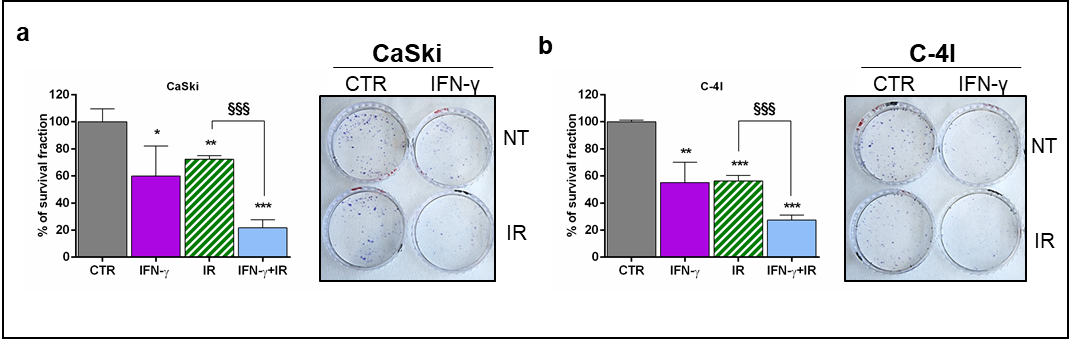
**

**Supplementary Fig. 2 Interferon-γ sensitizes radioresistant cervical cancer cells.** Clonogenic survival fractions of CaSki (**a**) and C-4I (**b**) cells treated with 10 ng/ml IFN-γ with or without IR (2 Gy). Cells were pre-treated for 2h with IFN-γ and then irradiated. Bar charts represent percentage of survival fraction normalized to CTR (mean ± SD, n=3). Representative pictures are shown on the right. Statistical significances have been evaluated through an unpaired t-test. **P*<0.05, ***P*<0.01 and ****P*<0.001 respect to CTR. §§§*P*<0.001 combined treatment with respect to IR. CTR: Untreated. IFN-γ: Interferon-γ.

**Supplementary Fig. 3**

**
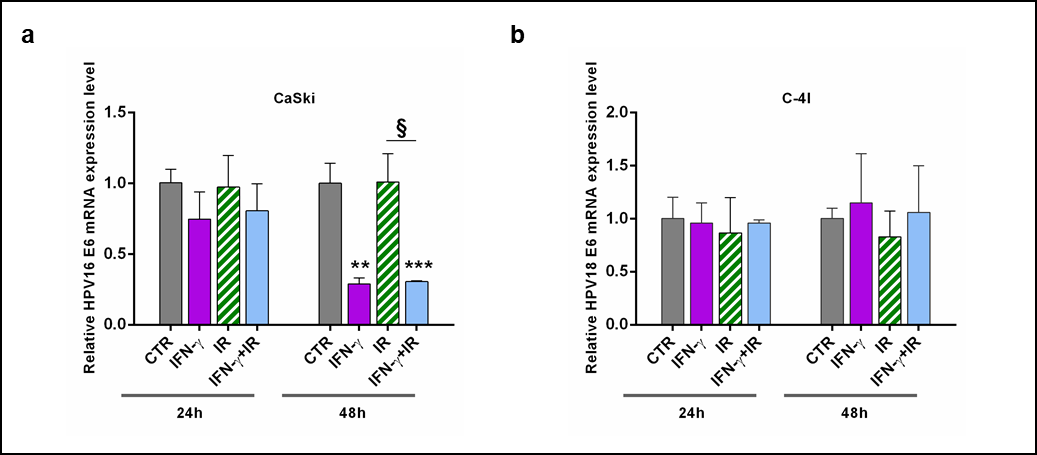
**

**Supplementary Fig. 3 Effects of Interferon-γ, with or without IR, on viral E6 mRNA in cervical cancer cells.** CaSki and C-4I cells were treated with 10 ng/ml IFN-γ (**a** and **b**) with or without IR (2 Gy). Relative viral E6 mRNA expression level was evaluated by RT-qPCR 24h and 48h after treatment. Results are presented as fold change compared to CTR cells (mean ± SD, n=2). Statistical significances have been evaluated through an unpaired t-test. **P<0.01 and ***P<0.001 respect to CTR. §P<0.05 combined treatment with respect to IR. CTR: Untreated. IFN-γ: Interferon-γ. IR: 2 Gy γ-rays.
